# Utilization of upper gastrointestinal tumor inpatient services affected by COVID-19 in China 2018–2021: an interrupted time series analysis

**DOI:** 10.3389/fpubh.2025.1589672

**Published:** 2025-10-01

**Authors:** Xue Yang, Mengying Liu, Hui Lu

**Affiliations:** Key Laboratory of Public Health Safety and Emergency Prevention and Control Technology of Higher Education Institutions in Jiangsu Province, Changzhou Institute for Advanced Study of Public Health, Nanjing Medical University, Nanjing, China

**Keywords:** interrupted time series, inpatient service, hospitalization costs, upper gastrointestinal tumors, blood supply, health equity, COVID-19

## Abstract

**Background:**

The COVID-19 pandemic has posed an unprecedented challenge to the healthcare system addressing chronic diseases, significantly affecting inpatient healthcare access. We aimed to determine the impact of COVID-19 on inpatient healthcare utilization for patients with upper gastrointestinal (UGI) tumors, which would help improve responses to medical healthcare needs under future public health emergencies.

**Method:**

Utilizing interrupted time-series analysis (ITSA), we analyzed data of UGI tumor patients from 37 medical institutions in Yangzhou from 2018 to 2021. Data were extracted from the Yangzhou City Health Information Platform in Jiangsu Province, and key indicators for analysis included demographics, clinical characteristics, and hospitalization utilization (length of hospital stay, hospital costs). The intervention point was set at January 2020, marking the onset of COVID-19.

**Result:**

Seven thousand three hundred and two cases were included in the analysis. The hospital days and total hospitalization costs decreased instantaneously by 1.60 (95%CI: −2.69, −0.51) days and 5349.04 (95%CI: −11015.66, 317.571) Yuan, respectively. During the post-pandemic period, hospital days and expenses surged, exceeding pre-pandemic levels by late 2021. The structure of medical costs has changed, with the cost of blood and consumables increasing by 345.53 (95%CI: 176.07, 514.99) Yuan and 755.23 (95%CI: −698.96, 2209.42) Yuan, respectively, at the time of the outbreak. Self-payment expense increased by 1150.77 (95%CI: –243.36,2544.91) Yuan, and reimbursement ratios slightly decreased by 0.03 (95%CI: –0.11, 0.05). Additionally, significant changes occurred in the occupational structure, health insurance utilization, and complication status of patients hospitalized with UGI tumors during the pandemic.

**Conclusion:**

Our findings indicate that the outbreak did cause a reduction in the use of UGI tumor inpatient services in Yangzhou City. The COVID-19 pandemic exacerbated the disease burden among patients with UGI tumors, with significantly increased costs for blood products and consumables. The findings emphasize the need to strengthen emergency management, implement precise prevention and control measures based on the dynamics of the epidemic, and ensure the safe supply of blood products and emergency medical supplies. It is necessary to improve the primary healthcare institution system to ensure patients’ access to medical services. The COVID-19 pandemic may exacerbate health inequalities. Therefore, it is essential to optimize the medical insurance system, provide targeted subsidies to medical institutions, curb the growth of unreasonable medical costs, and offer special protection for vulnerable groups.

## Introduction

China has a high disease burden of upper gastrointestinal (UGI) tumors, contributing almost half of the global incidence and deaths, with 478,000 (43.9% of the world) and 324,000 (53.7%) new cases of gastric and esophageal cancer, and 373,000 (48.5%) and 301,000 (55.7%) deaths in 2020 in China, respectively (1). Furthermore, the incidence of UGI cancer tends to be younger (2, 3), and tumors are more likely to be in middle or advanced stages when diagnosed (4). Patients with upper gastrointestinal tract cancer have a high demand for hospital services such as surgery and radiotherapy. The average length of stay for gastric cancer patients was 13.30 days, and for esophageal cancer patients was 13.90 days, according to China Health Statistics Yearbook 2022. The highly complex diagnostic and treatment processes impose a significant economic burden on the patients and their families (5). The mean UGI cancer inpatient costs ranged from 13,615.3 Yuan to 52,346.3 Yuan (6), and hospitalization costs as a percentage of annual household income exceed 40% (7). The utilization of medical services for patients with UGI tumors suffers from a long treatment period, high medical costs, and high demand for inpatient services.

Unaffordable medical costs, inconvenient transportation, lack of awareness, lack of trust in medical establishments, lack of coordination and incoherent referral pathways often contributed to the underutilization of UGI tumor inpatient services (8). Governments across China have launched a series of initiatives to reduce the burden of UGI cancer, including screening programs, additional reimbursement, clinical pathway management, level-by-level referral and others. However, access to UGI cancer treatment in China continues to be heavily impacted by the health-related inequity caused by various socio-economic factors. Narrowing international upper gastrointestinal tumor survival disparities, protecting the healthcare needs of UGI tumor patients and alleviating their financial burden is of vital importance and urgency.

China’s healthcare system adopts a multilevel framework, whose main components include primary healthcare (PHC) and secondary and tertiary hospitals. PHC serves as the frontline for preventive care, basic treatment and chronic disease management (9), with urban areas relying on community health centers and rural areas on township and village health centers and clinics (10); secondary and higher level hospitals deal with complex cases and referrals from PHC facilities and medical education (11). In an effort to facilitate the equitable distribution of medical resources, China has initiated the construction of a hierarchical diagnosis and treatment system (12). The hierarchical medical system involves primary medical institutions handling patients with minor illnesses, specialized hospitals treating patients with severe conditions, and community centers providing post-treatment rehabilitation services. It optimizes resource allocation by implementing a patient referral model (13). Research shows that the hierarchical medical system can significantly improve the diagnosis and treatment outcomes for patients with chronic diseases. It not only provides patients with more convenient daily health management but also reduces medical costs (14, 15).

There are three basic health insurance schemes in China: the Urban Employees Basic Medical Insurance (UEBMI), the Urban Resident Basic Medical Insurance (URBMI) and the New Rural Cooperative Medical Scheme (NRCMS) (16). China launched a comprehensive health-care reform in 2009 (17), establishing a basic health-care system that covers the entire population, with an overall coverage rate of 95 percent by 2019 (18). Despite China’s progress toward universal health coverage, the three basic medical insurance systems still vary markedly in funding and benefits (19). In addition, since 2005, China has supported population-based screening and early diagnosis and treatment of UGI cancer in areas with high incidence of UGI tumors in the form of central financial transfer payments (20). The program provides screening for UGI cancer for people aged 40–69 in areas with a high incidence of the disease (21, 22). By the end of 2020, the screening coverage had already covered 31 provincial administrative regions, with more than 2.6 million people of the right age screened and the early diagnosis rate reaching 70% (20, 23).

The COVID-19 pandemic has become a major challenge for all health care systems worldwide. Medical institutions struggle to balance the priorities of COVID-19 prevention and control, while also ensuring that essential public health services is safe and accessible. Studies have demonstrated that healthcare utilization across the world decreased by about a third during the pandemic, with a median 37% reduction in services overall with considerable variation, comprising median reductions for visits of 42% and admissions 28% (24, 25). While coping with a public health threat, an ill-resourced health system tends to sacrifice access to essential health services for chronic patients (26). The availability and maintenance of cancer services were substantially affected by the pandemic. Medical visits, surgeries, radiotherapy and chemotherapy sessions were interrupted or delayed due to subjective and objective factors (27, 28). Cancer patients in the United States, South Korea and other countries had to cancel or delay cancer care due to COVID-19 (29, 30). The prevalence of Covid-19 has also disrupted long-term screening programs that prevent diseases or can lead to early detection of UGI cancer (31, 32).

China has been actively fighting to control the COVID-19 epidemic. Since the epidemic outbreak to early 2020, all the 31 provincial-level regions in the Chinese mainland had initiated a first-level response (33, 34). China implemented strict comprehensive prevention and control measures, such as traffic restrictions, home quarantine, and large-scale nucleic acid testing (35). While these policies effectively prevent the spread of COVID-19, they also hurt patients’ ability to receive timely care (36). Many provinces and cities in China have explored the impact of COVID-19 on medical services (37, 38). The utilization of all types of healthcare services including UGI cancer inpatient services declined markedly after the outbreak of COVID-19 (39).

Studies have demonstrated that COVID-19 has caused extensive economic losses, leading to significant disruptions in daily life and healthcare delivery in China (40). However, few studies have focused solely on service utilization in upper gastrointestinal tumors. In addition, most studies have explored inpatient service utilization through changes in variables such as the number of visits, length of stay. However, to date, hospitalization costs, particularly specific categories of hospitalization costs such as diagnostic fees and drug costs, are rarely used in studies of UGI cancer inpatient service utilization.

Yangzhou City has a traditional high incidence of upper gastrointestinal tract tumors in China, with a resident population of 4,571,400 and a proportion of 26.00 per cent of the population aged 60 and over. Gastric cancer and oesophageal cancer ranked second and third in the cause of death of malignant tumors of Yangzhou residents, with a death standardization rate of 49.80 and 41.61 per 100,000 people, respectively, (41). Yangzhou city has a high disease burden of UGI tumors and a high demand for inpatient services among residents, which can better reflect the impact of COVID-19 on the utilization of inpatient healthcare services, and has a good representation. Therefore, in this study, we analyzed the variation in inpatient days and inpatient costs for UGI cancer patients from 2018 to 2021 in Yangzhou City, using interrupted time series analysis (ITSA). The objective of our study is to figure out how public health emergencies would affect the utilization of UGI tumor inpatient services and how interventions should be implemented to improve accessibility and availability in the quality of UGI cancer care.

## Methods

### Data sources

We obtained data from the Yangzhou City Health Information Platform in Jiangsu Province, which includes 9 level II and above hospitals and 28 level I hospitals. We collected case data information from January 1, 2018, to December 31, 2021, from the Western Medical Case Home Form for inpatient UGI tumor patients with a primary diagnosis of esophageal cancer (ICD-10: C15.0-C15.9) and gastric cancer (ICD-10: C16.0-C16.9).

The database included demographic information (i.e., patient’s age, gender, nationality, residence and residence code), hospitalization information (i.e., disease name and code, length of hospital stay and hospital costs). Abnormal data, including duplicate records, missing information on the composition of hospital costs and outliers, were excluded. Information on 7,302 cases was included in the analysis.

This study focuses on the utilization of UGI tumor inpatient services affected by emergencies. Thus, interrupted time series analysis was used to evaluate the immediate and long-term trends in hospital days and hospital costs of UGI oncology patients in Yangzhou before and after the epidemic. It is important to note that ITSA can reveal temporal associations between interventions and outcomes, but cannot determine causality.

### Variables and outcomes

The core variables are the number of hospital days and the amount of hospital costs per month. The subvariable variables are total hospitalization costs’ specific composition (seven categories, including treatment costs, drug costs, service costs, diagnostic costs, blood costs, consumables costs and other costs), self-payment and reimbursement ratio per month. To control for the effects of inflation and price changes on the 4-year comparison of hospitalization costs, the costs for 2018–2020 were adjusted according to the Consumer Price Index (CPI) for Yangzhou City based on the 2021 level. The selection of 2021 as the baseline year ensured all costs were expressed in the most recent currency values, minimizing temporal biases while reflecting post-pandemic economic conditions.

Taking into account the time of the COVID-19 outbreak and the implementation of the Level 1 public health emergency response in Yangzhou City (26 January 2020), this study selected January 2020 as the intervention time point, after which public transport in Yangzhou City was suspended and people’s access to health care was restricted. And the entire observation period was divided into the pre-pandemic (pre-intervention) period (January 2018–December 2019) and the post-pandemic (post-intervention) period (January 2020–December 2021), to establish the following analytical model.

### Interrupted time series analysis (ITSA) for single group

Using Stata 17.0 software, we included the inpatient days and inpatient costs per month from 2018 to 2021 in an ITSA for singlegroup.


$$Y_{t}=\beta_{0}+\beta_{1}\times \text{time}+\beta_{2}\times \text{intervention}+\beta_{3}\times \text{post}+\varepsilon_{t}$$

$Y_{t}$ denotes the observation of the time series. *time* denotes the time series, *time* = 0, 1, 2,…, n-1, where n is the number of observation points. *intervention* denotes the intervention phase in which the observation point is located, the observation point before the intervention takes the value of 0, and the observation point after the intervention takes the value of 1. *post* denotes the time series after the intervention, the observation point before the intervention *post* takes the value of 0, the first observation point after the intervention *post* takes the value of 1, and so on. $\;\varepsilon_{t}$ denotes the random error independent of time. The parameters were obtained by fitting a regression model: baseline level $\;\beta_{0}$ ; pre-intervention slope $\beta_{1}$ ; instantaneous level change $\beta_{2}$ ; and slope change $\beta_{3}$ , which is the difference between the post-intervention slope and the pre-intervention slope.

The Durbin-Waston test was used for auto-correlation, and when auto-correlation existed, the bias due to auto-correlation in the regression was dealt with using the Newey-West method at a test level of *α* = 0.05.

## Results

### Descriptive statistics

In this study, we collected 7,302 hospitalized cases of upper gastrointestinal tumors. Esophageal cancer contributes to almost 35% of the total cases, and gastric cancer to about 65%. Table 1 presents the characteristics of inpatients with UGI cancer from 2018 to 2021. Male patients accounted for more than 70% and more than 80% of the population was aged 60 years or older across all periods. Compared with the pre-pandemic period, the percentage of workers, farmers, retirees and patients without jobs was lower, while the ratio of patients engaged in other professions rose by 19.98 percentage points during the post-pandemic period. Between the pre-pandemic period to the post-pandemic period, the proportion of patients hospitalized with UGI cancer using health insurance for urban employees increased, while the proportion using health insurance for urban and rural residents and self-pay decreased. There was a slight increase in the proportion of hospitalized patients undergoing surgery during the pandemic. The group of hospitalization days composition ratios were, in descending order, 11–20 days, 1–10 days, 21 to 30 days and greater than 30 days in 2018–2021.

**Table 1:** Description of inpatients with upper gastrointestinal tumors from 2018–2021(%).

Characteristic	2018–2019 (*N* = 4,341)	2020–2021 (*N* = 2,961)	χ^2^	*p*
Gender			0.288	0.591
Male	3,133(72.17)	2,120(71.60)		
Female	1,208(27.83)	841(29.11)		
Age			1.344	0.511
<45	58(1.34)	49(1.65)		
45–59	663(15.27)	459(15.50)		
≥60	3,620(83.39)	2,453(82.84)		
Marital status			20.003	**<0.001**
Married	4,090(94.22)	2,709(91.49)		
Others	251(5.78)	252(8.51)		
Occupational type			398.919	**<0.001**
Office worker	108(2.49)	64(2.16)		
Worker	246(5.67)	48(1.62)		
Farmer	1,636(37.69)	825(27.86)		
Retiree	724(16.68)	356(12.02)		
Unemployed	109(2.51)	41(1.38)		
Others	1,518(34.97)	1,627(54.95)		
Health insurance schemes			408.256	**<0.001**
UEBMI	1,264(29.12)	995(33.60)		
URBMI	1946(44.83)	943(31.85)		
NRCMS	222(5.11)	74(2.50)		
No health insurance	455(10.48)	180(6.08)		
Others	454(10.46)	769(25.97)		
Hospital level			0.624	0.430
Level II and above	4,173(96.13)	2,856(96.45)		
Level I	168(3.87)	105(3.55)		
Operation			6.337	0.012
Yes	2,915(67.15)	2071(69.94)		
No	1,426(32.85)	890(30.06)		
Hospital days			5.127	0.163
1–10	1,341(30.89)	939(31.71)		
11–20	2,162(49.80)	1,469(49.61)		
21–30	637(14.67)	391(13.20)		
>30	201(4.63)	162(5.47)		
Co-morbidity			93.947	**<0.001**
Yes	1,464(33.72)	1,330(44.91)		
No	2,877(66.28)	1,631(55.09)		

Bold values indicate statistical significance, with *p* < 0.05.

**Figure 1:**
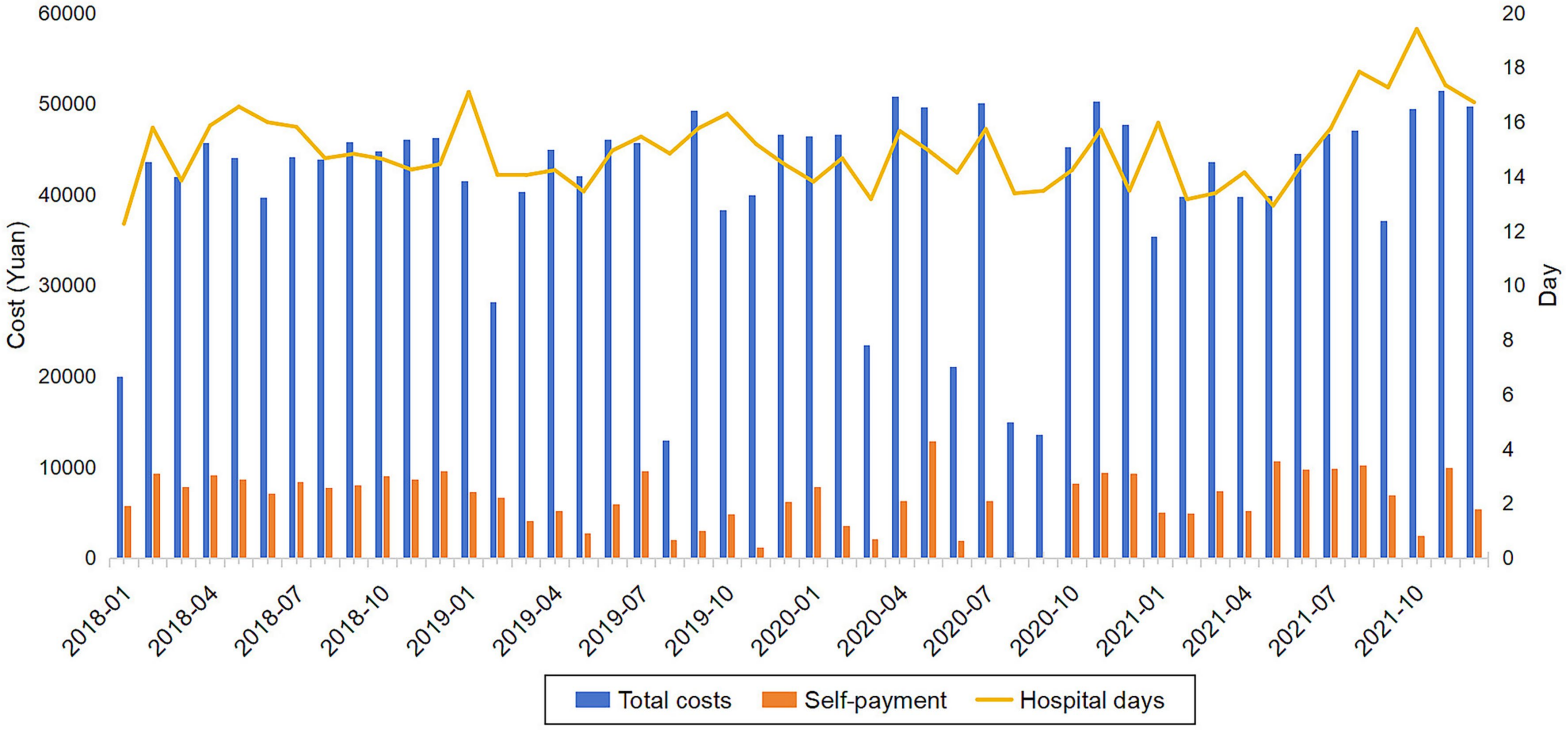
Number of hospital days, total hospitalization costs and self payment burdun per month for patients with UGIl tumors from 2018 to 2021.

Table 2 shows the inpatient days, inpatient costs and specific inpatient costs for patients with upper gastrointestinal tract tumors before and during the pandemic. The average cost of hospitalization for patients was 42,899.46 Yuan, and the average number of days in hospital was 14.90 days before the pandemic and 46,468.88 Yuan and 14.75 days after the pandemic, respectively. The self-paid medical expenses also increased slightly with the increase of hospitalization costs, increasing by 61.54 Yuan. The number of hospital days, total hospitalization costs and self-payment burden per month for patients with upper gastrointestinal tumors from 2018 to 2021 are also depicted in Figure 1.

**Table 2:** Hospitalization days, hospitalization costs and their components for patients with upper gastrointestinal tumors from 2018 to 2021.

Variable	2018–2019 (*N* = 4,341)	2020–2021 (*N* = 2,961)	Percent change
Hospitalization days	14.90	14.75	−1.01
Hospitalization cost			
Total	42899.46	46468.88	8.32
Self-pay	6792.33	6853.87	0.91
Drug	10751.28	10238.75	−4.77
Consumables	12181.10	12730.86	4.51
Diagnostic	5781.16	6239.10	7.92
Treatment	6496.51	6303.0828	−2.98
Service	3245.19	3064.16	−5.58
Blood	249.40	542.77	117.63
Others	249.16	431.27	73.09
Reimbursement ratio	0.80	0.79	−1.25

The composition of hospitalization costs for patients with UGI tumors changed somewhat at different stages of the pandemic. While the treatment fee had not changed much over the pandemic period, the blood fee had shown an increasing trend year by year. The largest increase was in the blood fee (117.63%), which rose from 249.40 Yuan to 542.77 Yuan, and the largest decrease was in the service fee (−5.58%), which fell from 3245.19 Yuan to 3064.16 Yuan. After the outbreak, the cost structure is consumables fee, drug fee, treatment cost, diagnostic fee, service cost and blood fee.

### Interrupted time series analysis facts

Table 3 shows the ITSA results for inpatient days, inpatient costs, specific hospitalization costs, and reimbursement ratio. These findings constitute the primary evidence for assessing the causal impact of the COVID-19 pandemic.

**Table 3:** Impact of COVID-19 on cost components of hospitalization for patients with upper gastrointestinal tract.

Variable	Intercept		Pre-intervention slope		Instantaneous level change		Slope change	
	Coefficient (95% CI)	*p*-value	Coefficient (95% CI)	*p*-value	Coefficient (95% CI)	*p*-value	Coefficient (95% CI)	*p*-value
Hospitalization days	14.83 (14.38, 15.28)	<0.001	0.01 (−0.02,0.04)	0.491	−1.60 (−2.69,-0.51)	**0.005**	0.12 (0.03,0.22)	**0.012**
Hospitalization cost								
Total	40578.78 (37264.71,43892.85)	<0.001	25.484 (−220.58,271.55)	0.836	−5349.04 (−11015.66,317.571)	0.064	421.53 (59.04,784.03)	**0.024**
Self-pay	9117.12 (8081.15, 10153.10)	<0.001	−223.77 (−303.51,-144.03)	**<0.001**	1150.77 (−243.36,2544.91)	0.103	358.22 (254.22,462.22)	**<0.001**
Drug	11044.69 (10445.36,11644.02)	<0.001	−24.37 (−68.61, 19.87)	0.273	−1219.68 (−2871.90,432.54)	0.144	99.78 (−9.67,209.24)	0.073
Consumables	11048.55 (9715.14,12381.97)	<0.001	79.23 (−13.95, 172.41)	0.094	755.23 (−698.96, 2209.42)	0.301	−133.10 (−255.52,-10.69)	**0.034**
Diagnostic	5601.79 (5030.07, 6173.51)	<0.001	−4.07 (−49.20,41.05)	0.856	−396.79 (−1190.92,397.32)	0.319	67.65 (19.30,115.99)	**0.007**
Treatment	6027.26 (5228.2,6826.31)	<0.001	3.85 (−59.60,67.31)	0.903	−942.69 (−2509.17, 623.79)	0.232	30.36 (−72.90, 133.62)	0.557
Service	3429.29 (3127.28,3731.31)	<0.001	−31.77 (−54.96,-8.58)	**0.008**	−281.58 (−740.61,177.45)	0.223	70.93 (42.43, 99.42)	**<0.001**
Blood	104.47 (73.96,134.98)	<0.001	11.28 (8.53, 14.02)	**<0.001**	345.53 (176.07,514.99)	**<0.001**	−20.31 (−34.95, −5.68)	**0.008**
Others	217.13 (−40.78,475.03)	0.097	11.94 (−7.91, 31.78)	0.232	37.66 (−295.06, 370.39)	0.821	−18.56 (−40.78, 3.66)	0.099
Reimbursement ratio	0.74 (0.68,0.78)	<0.001	0.00 (0.00,0.01)	**0.028**	−0.03 (−0.11,0.05)	0.526	−0.01 (−0.01,0.00)	**0.032**

Bold values indicate statistical significance, with *p* < 0.05.

**Figure 2:**
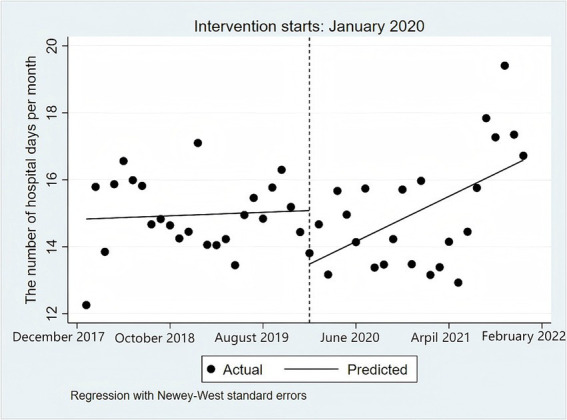
ITSA results for hospital days among patients with UGIl tumors from 2018 to 2021.

The corresponding curve of hospitalization days is also depicted in Figure 2. Before the pandemic, hospitalization days exhibited a generally stable trend with minor fluctuations. When the pandemic broke out, the number of hospitalization days fell to a low point. Figure 2 vividly demonstrates an abrupt decrease at the intervention point in hospital days. The average patient hospitalization days decreased by 1.60 (95%CI: −2.69, −0.51) days at the moment of the outbreak. During the post-pandemic period, the number of average patient hospitalization days had an upward trend with a slope of 0.12 (95%CI: 0.03,0.22) days per month. In January 2021, the number of hospital days had nearly risen to historical levels before the pandemic. In the last few months of 2021, the average length of hospital stay for patients with upper gastrointestinal cancer was significantly higher than the average level, showing a rebound trend.

The ITSA results showed that the impact of the COVID-19 pandemic on specific hospitalization costs was inconsistent, with some going up, some going down, and other changes not significant enough to be statistically significant. Pre-pandemic patient diagnostic fees showed a slight decreasing trend, decreasing by 4.07 (95%CI: −49.20,41.05) Yuan per month. Despite a significant drop in diagnostic fees at the time of the outbreak, during the pandemic period, patient diagnostic fees changed from a decreasing trend to an increasing trend, increasing by an average of 67.65 (95%CI: 19.30, 115.99) Yuan per month. The blood fee of patients increased by 345.53 (95%CI: 176.07, 514.99) Yuan instantaneously in January 2020. As the pandemic was brought under control, the cost of blood should have been effectively reduced. However, the result shows that although the blood cost showed a downward trend after the outbreak, the blood fee during the post-pandemic period was still higher than that before the pandemic. It is worth noting that at the moment of the outbreak, the amount of self-payments has increased by 1150.77 (95%CI: −243.36, 2544.91) Yuan, and its post-pandemic growth is higher than pre-pandemic growth. Consumables fee grew rapidly at the outbreak, increasing expenses have reached a staggering 755.23 (95%CI: −698.96, 2209.42) Yuan. However, it showed a downward trend during the post-pandemic period. Changes in the drug cost, treatment cost and other costs were not significant.

## Discussion

The outbreak has had a significant impact on service utilization for patients with upper gastrointestinal tumors. At the beginning of the pandemic outbreak, the average number of hospitalization days for patients with UGI tumors dropped by 1.60 (95%CI: −2.69, −0.51) days (*p* < 0.05) immediately. In the early stages of the pandemic, the use of medical services was reduced due to multiple factors such as policy and hospital emergency response capacity. Firstly, policies can significantly affect people’s medical service utilization behavior (42, 43). Yangzhou city has officially activated the first-level response to public health emergencies on 26 January 2020. Subsequently, rigorous public health interventions were implemented, and the public transportation system, such as buses, taxis, was suspended in the main urban area. And hospitals have established a series of regulations, including staying in transitional wards, prohibiting visits and limiting the number of escorts (44). These measures imposed during the pandemic made it difficult for patients with UGI tumors to travel to medical institutions to access care and have reduced patients’ willingness to be hospitalized and artificially reduced their length of stay.

Secondly, healthcare providers are unable to maintain daily routines during times of outbreaks. The additional burden of treating COVID-19 patients and the loss of personnel due to COVID-19 infection have combined to reduce the provision of medical services including medical visits, surgeries, procedures, and radiotherapy and chemotherapy sessions. Transportation restrictions and increased demand for medical protective equipment together have led to medicine shortages and other health product shortages including devices, personal protective equipment (PPE), and laboratorial or image tests. Owing to the shortage of doctors and medical supplies, patients have to postpone or suspend the utilization of in-patient services, such as elective surgeries and regular chemotherapy.

Finally, patient factors leading to decreased utilization. A series of studies reported that the fears of infectious diseases significantly influenced people’s care-seeking behavior and compromised their accessibility to quality care (45, 46). The Chinese Center for Disease Control and Prevention (CDC) has publicly reported the daily pandemic infection situation and virus research progress to the whole country through various media, so as to make the pandemic information open and transparent. However, fears of emerging infectious diseases cannot be easily dismissed. We still need to work to prevent people from delaying access to the hospital out of fear, which may result in more serious health consequences.

Since September 2021, Yangzhou’s medium-risk areas have been cleared, urban traffic has basically returned to the pre-pandemic level, the demand for patient consultation and treatment has been fully released, and the hospital days have subsequently risen. With the liberalization of the pandemic restriction policy, inpatient admissions began to go back to historical levels. The number of days of hospitalization shows an upward trend, increasing by 0.12 (95%CI: 0.03,0.22) days (*p* < 0.05). In December 2021, the average hospitalization days of patients were 16.72 days, far exceeding the pre-pandemic level, a phenomenon consistent with most studies on policy affecting the utilization of inpatient health services (47, 48). This may indicate that that the supply of medical services was significantly constrained during the epidemic, with routine diagnostic and treatment services forced to be interrupted due to prevention and control measures and other reasons, and that the suppressed demand for medical services was not adequately met, which manifested itself in a significant lengthening of hospitalization in the latter stages of the epidemic. It suggests that the relevant departments need to improve the emergency response mechanism for public health emergencies in their daily work. During the pandemic prevention and control period, policies based on real-time pandemic status should be introduced to support patients’ access to primary health care and to safeguard their basic diagnostic and treatment needs. Patients should be made fully aware that the risks of delayed access to hospital care could be much higher than those posed by COVID-19. In the event of an outbreak, the national health commission should soothe public sentiments, devising and implementing interventions so that all patients feel safe seeking the care (49). Some studies have shown that a well-established system of primary health-care facilities, with adequate support for community health workers, financial remuneration, training, supplies and supervision in accordance with WHO guidelines, may mitigate the impact of health-system shocks (50). For instance, in the early stage, timely formulation of policies to facilitate access to medical care, stratified and classified management of patients, and in the late stage, smooth “two-way referral” channels, so as to be vigilant about the rebound of the number of days of inpatient (51).

Utilization of inpatient services has a direct impact on hospitalization expenses (52). Due to the reduction in utilization of inpatient services, the total cost for patients with UGI tumors decreased instantaneously by 5349.04 (95%CI: −11015.66, 317.571) Yuan (*p* > 0.05) at the time of the outbreak. In the last few months of 2021, the total hospitalization costs increased rapidly because of the significant demand for inpatient service utilization, and the growth rate exceeded the growth expectation. Compared with the pre-pandemic period, total medical expenses increased significantly by 421.53 (95%CI: 59.04,784.03) Yuan (*p* < 0.05) post-pandemic, with a markedly higher growth rate than before the pandemic. While most previous studies have focused only on the association between inpatient service utilization and total costs, this study delves into the changes in the specific hospitalization costs, the reimbursement ratio and the occupational structure, health insurance use, and co-morbidity status of hospitalized patients during the pandemic.

The total hospitalization costs decreased due to shortened length of stay, while patients’ self-payment expenses increased by 1150.77 (95%CI:–243.36,2544.91) Yuan. Although this difference was not statistically significant (*p* > 0.05), it still suggests that during the pandemic period, we need to pay attention to maintaining a balance between patients’ actual financial burden and healthcare accessibility. The average self-payment expenses of inpatients with upper gastrointestinal cancer increased from 6792.33 Yuan before the outbreak to 6853.87 Yuan. Pre-pandemic data showed a significant decrease in self-payment expenses by −223.77 (95%CI: −303.51,-144.03) Yuan (*p* < 0.001), while a post-pandemic significant increase of 358.22 (95%CI: 254.22,462.22) Yuan (*p* < 0.001) was observed. This clear trend strongly supports our research hypothesis that the COVID-19 pandemic has exacerbated the financial burden on hospitalized UGI cancer patients. This inference aligns with prior research demonstrating that COVID-19 leads to higher healthcare expenditures among chronic disease patients (53). We speculate that the reason for this may be related to changes in reimbursement rates. The health insurance reimbursement ratio remained stable before the pandemic with a change value of 0.00 (95%CI: 0.00, 0.01) (*p* < 0.05), and then demonstrated a significant downward trend post-pandemic, decreasing by 0.01 (95%CI:–0.01,0.00) (*p* < 0.05). These changes indicate that the pandemic significantly affected reimbursement rates, consequently leading to increased self-payment expenses by patients.

At the beginning of the pandemic outbreak, the patient blood fee increased by 345.53 (95%CI: 176.07, 514.99) Yuan (*p* < 0.001) immediately. With the epidemic being brought under control, there has been a downward trend in blood fees in the later stage of the epidemic, with a decrease of 20.31 (95%CI: −34.95, −5.68) Yuan (*p* < 0.05). This dynamic adjustment process reflects the resilience of China’s medical system. It is worth noting that the increase in the patient blood fee during the whole pandemic reached 117.63%, and the current cost of blood testing has not yet fully recovered to the pre-epidemic level, which may be related to the continued tight supply chain of blood products. COVID-19 poses serious challenges to the global blood supply, including loss of key personnel, disruptions in the supply chain and initial uncertainty about the safety of blood transfusions (54, 55). The decline in the number of donors has led directly to a shortage of blood products (56). These factors led to soaring blood costs during the pandemic, adding to the financial burden on patients. China has basically formed a nationwide blood station service system. However, under the national and global public health events such as COVID-19, China’s blood emergency mechanisms still face challenges. National Blood Center and other related institutions should continue to ensure the safety and supply of raw plasma and blood products, providing patients with safe, reliable and affordable blood products. Strengthening blood inventory management and dynamic monitoring, providing timely early warning, and ensuring plasma sufficiency are the basis for the orderly implementation of all diagnostic and therapeutic work in response to public health events (57).

Medical consumables and drugs have always been a major component of hospitalization costs, and policies related to cost control are a hot topic in current research. The results of this study show that drug cost control has been effective. The pandemic did not lead to an increase in drug costs. Even though the drug costs increased during the epidemic period, the overall trend was stable and has returned to normal levels, which is consistent with other studies (58). However, the consumables cost increased by 755.23 (95%CI: −698.96, 2209.42) Yuan (*p* > 0.05) as total hospitalization costs decreased due to the outbreak. Even in the post-pandemic period, the cost of consumables was gradually reduced by 133.10 (95%CI: −255.52, −10.69) Yuan (*p* < 0.05). However, by December 2021, the cost level still had not returned to the pre-pandemic baseline. The outbreak of COVID-19 has led to an explosive increase in the cost of consumables, and this phenomenon deserves our utmost attention.

On the one hand, the COVID-19 pandemic has exponentially escalated the demand for medical consumables. All patients and healthcare workers must use certified protective equipment, and patients are subject to mandatory preoperative nucleic acid detection according to infection control measures/protocols (59). The global shortage of PPE during the first wave of the pandemic (60), coupled with rising raw material prices, which resulted in increasing costs for patient consumables. The establishment of a better store of health emergency supplies is critical for service utilization during epidemics (61). On the other hand, this may also be related to the fact that the hospital faced financial challenges during the epidemic due to a reduction in hospital visits and a lack of subsidies for COVID-19 (62, 63). Thus, hospitals may have been compelled to implement measures to compensate for the loss to maintain basic hospital operations, such as raising revenue from consumables (64, 65). Research indicates that following the first wave of COVID-19 in 2020, total hospitalization costs experienced a growth of approximately 8.7 to 16.7%, which was largely driven by an increase in the expenses for laboratory tests and medical consumables (66). To ensure the sustainable operation of healthcare institutions, relevant departments should provide subsidies to healthcare institutions for the loss due to admitting COVID-19 patients and inability to carry out normal diagnostic and treatment activities as a result of outbreak-related policies.

In addition to the significant changes in blood costs and consumable costs, it is noteworthy that diagnostic fees and service fees also showed a slight upward trend during the pandemic. This phenomenon may be related to the adjustment of the operation models of medical institutions during the pandemic. The diagnostic fees exhibited a declining trend before the COVID-19 pandemic, with a decrease of 4.07 (95%CI: −49.20, 41.05) Yuan (*p* > 0.05). The outbreak of the pandemic led to a decrease of 396.79 (95%CI: −1190.92, 397.32) Yuan (*p* > 0.05). In the later stage of the pandemic, the diagnostic fees showed an upward trend, with an increase of 67.65 (95%CI: 19.30, 115.99) Yuan (*p* < 0.05). And the service fee showed a downward trend before the COVID-19 pandemic, decreasing by 31.77 (95%CI: −54.96, −8.58) Yuan (*p* < 0.05). At the onset of the COVID-19 pandemic, service fees decreased sharply by 281.58 (95%CI: −740.61,177.45) Yuan (*p* > 0.05), while in the post-pandemic period, it rebounded with an increase of 70.93 (95%CI: 42.43, 99.42) Yuan (*p* < 0.001).

Before the outbreak of the COVID-19 pandemic, under the combined effect of the public hospital reform and informatization construction, China’s medical system significantly improved its diagnosis and treatment efficiency, and service costs gradually decreased (67, 68). However, in the initial stage of the pandemic outbreak, strict prevention and control measures led to restrictions on inpatient services (69). The total inpatient costs decreased by 5,349.04 (95% CI: −11,015.66, 317.57) Yuan in the short term. Diagnostic fees and service fees also decreased simultaneously due to the reduction of inspection items and the limitation of basic nursing services. In the middle and later stages of the pandemic, with the gradual restoration of the diagnosis and treatment order, multiple factors, including the increase in medical costs caused by the pandemic, the upgrading of medical technologies and equipment, and the lack of subsidies for hospitals (70, 71), led to an increase in costs. This fluctuating trend indicates the sensitivity of diagnostic fees and service fees during public health emergencies. It is necessary to improve the flexible pricing mechanism to better control inpatient costs.

In addition to the impact of the COVID-19 pandemic, it is crucial to consider the concurrent implementation of the Diagnosis-Related Groups (DRG) payment reform as a significant confounding factor. This policy could independently influence hospitalization costs and length of stay by incentivizing shorter stays and more cost-effective resource use. Our study period overlaps with the early stages of DRG implementation in some areas. Therefore, the observed reductions in length of stay and changes in cost structure may partly reflect the effects of DRG policies rather than solely the pandemic. Future studies should seek to disentangle these overlapping policy impacts to more accurately assess the independent effect of public health emergencies on healthcare utilization.

Furthermore, the findings of this study suggest that the COVID-19 pandemic may have exacerbated disparities in healthcare utilization among upper gastrointestinal cancer patients, a concerning trend that warrants heightened attention. The occupations of patients with UGI cancer are predominantly workers, farmers, retirees, and other people with relatively low economic levels. The data showed a significant change in the occupational distribution of patients (*p* < 0.001) during the epidemic. The proportion of farmers decreased from 37.69 to 27.86%, retirees from 16.68 to 12.02%, while the other occupational category increased significantly from 34.97 to 54.95%. This change may be due to the impact of the epidemic on the labor market: farmers and retirees reduced their access to health care due to the risk of infection or travel restrictions, while the increase in the proportion of other categories, such as freelancers and flexibilizers, may be related to the increase in the unemployment rate during the epidemic or to career changes. Compared with patients with better economic conditions, the inability to obtain affordable and convenient transportation to seek medical treatment may have a greater impact on the utilization of services for such patients, resulting in greater medical inequality during the pandemic. The popularity of online healthcare should be accelerated to meet the needs of patients for inpatient consultation and treatment when patients are unable to go to medical institutions for various reasons (72, 73). Existing studies have shown the least increase in telemedicine service utilization during the outbreak in the older age group ≥65 years (74), which happens to be highly overlapping with the main population of patients hospitalized with UGI tumors. Considering the low level of acceptance and utilization of telemedicine by older and lower-income inpatients, there is a need for health education campaigns and case-by-case guidance on the use of online healthcare for older adults on a daily basis. Pay attention to and effectively carry out daily health science popularization and health education work, so that patients and their families can enhance the initiative and enthusiasm for self-health management.

China has achieved universal social health insurance coverage, but there are still discrepancies between urban and rural areas in terms of the impact of health insurance (75). It is unclear whether the disease burden of people with different types of medical insurance can be effectively alleviated when public health emergencies occur. During the pandemic, a notable change in the payor mix for upper gastrointestinal tumor patients occurred (*p* < 0.001), marked by a decline in self-pay, urban, and rural resident insurance cases. According to Chin’s medical insurance policy, the reimbursement rates of UEBMI, URBMI, and NRCMS were 72, 50, and 40%, respectively (76). Moreover, as a result of the epidemic, it is likely that the URBMI and NRCMS fund will suffer a current deficit, which may affect the well-being of participants, while UEBMI will retain a considerable surplus (77). Compared with UEBMI, the URBMI and the NRCMS have lower reimbursement rates and higher out-of-pocket payments, which means that insured patients have to bear a heavier financial burden. Research shows that the pandemic would reduce household per capita income by 8.75% for rural residents and 6.13% for urban residents (78). The patients who have a lower reimbursement ratio insurance or even no medical insurance may have reduced their utilization of health services due to financial problems. It is also worth noting that other types of health insurance, which include commercial insurance, rose from 10.46 to 25.97%, suggesting that the epidemic has prompted some of the population to seek supplementary coverage (79, 80). Studies have also shown that major public health emergencies can increase commercial insurance participation, but the promotion of commercial insurance for rural and low-income populations is relatively limited (81).

In addition, the co-morbidity rate increased significantly from 33.72 to 44.91% (*p* < 0.001). This may be due to two reasons: firstly, the epidemic prevention and control measures have led to a serious impact on the routine medical care and follow-up of patients with chronic diseases (82). Patients with chronic diseases requiring long-term management, such as hypertension and diabetes, have seen their underlying disease control deteriorate significantly as a result of delays in seeking medical care (83); secondly, the COVID-19 infection itself may induce or exacerbate co-morbidities, especially respiratory and cardiovascular diseases (84, 85). Furthermore, factors such as psychological stress and lifestyle changes brought about by the epidemic may influence the development of co-morbidities (86). This phenomenon suggests that we need to pay special attention to the impact of public health emergencies on the health management of patients with chronic diseases.

Research suggests that vulnerable groups, particularly low-income and older adults (87), may experience greater challenges during a pandemic, raising concerns that healthcare disparities in China’s inpatient services may widen. Changes in occupational distribution suggest that low-income groups may have barriers to healthcare utilization, healthcare insurance data reveals coverage imbalances in current healthcare policies, and increasing co-morbidity rates highlight the significant impact of public health emergencies on chronic disease management. Notably, the elevated number of self-pay medical expenses persisted after the outbreak, emphasizing the need for the state and society to pay attention to the economic problems of vulnerable groups for medical treatment. Therefore, It is suggested that future policies need to enhance access to health care for vulnerable occupational groups and the government should optimize health insurance systems by increasing overall capital investment and financial support for vulnerable groups such as rural population and poor population to reduce the economic burden and improve the equity of distribution of healthcare benefits, and improve mechanisms for emergency management of chronic diseases.

The financial burden of UGI cancer patients is already significant, and every effort should be made to reduce unreasonable costs to safeguard the need for medical treatment. On the premise of ensuring the effectiveness of treatment, the relevant medical institutions should curb the unreasonable increase in hospitalization costs and optimize the structure of medical costs, in accordance with the implementation plan of universal health coverage. Sufficient compensation from medical services and government subsidies that minimize the income effects may be the key to the success of price change reform (88). Government needs to take a leading role to improve the performance appraisal system orientated toward public welfare and based on public welfare, health output, and service quality.

In this study, we employed the ITSA method to analyze the impact of COVID-19 on service utilization among inpatients with upper gastrointestinal tumors, and propose measures to better safeguard oncology services in the event of public health emergencies in the future. However, it must be clearly pointed out that although ITSA can reveal the association between interventions and research outcomes in the time dimension, it cannot directly establish a causal relationship between the two. The observed changes in the length of hospital stay and the structure of hospitalization costs may be simultaneously influenced by external factors. These include broader economic turmoil in early 2020 (89), parallel health policy reforms such as adjustments to insurance reimbursement rates, and the implementation of the Diagnostic - Related Groups payment pilot reform (90).

Some limitations of our study must be acknowledged. Firstly, our analysis was restricted to data from Yangzhou City, which is characterized by an economically developed city with a high incidence of UGI tumors, which may limit the broader applicability of findings to other regions, such as less developed regions or areas with lower UGI tumor incidence rates. What’s more, the city’s well-established healthcare infrastructure and relatively affluent population may not be representative of less developed or rural regions in China, where healthcare resources are scarcer and transportation barriers are more pronounced. In such areas, the impact of the COVID-19 pandemic on healthcare utilization and financial burden could be even more severe due to pre-existing vulnerabilities. Second, the study did not stratify for different UGI tumor types (oesophageal versus gastric cancer) and tumor stage or treatment modalities (surgical versus non-surgical), and such subgroup analyses may reveal potential differences in healthcare utilization. Third, although a significant increase in comorbidity rates was observed during the epidemic, the lack of detailed data on the severity and specific types of comorbidities prevented us from accurately determining whether this phenomenon stemmed from delays in the treatment of chronic illnesses, the direct impact of COVID-19 infection, or a combination of both.

To better explore the impact of the epidemic on service utilization, we recommend future multicenter studies covering different geographic characteristics and levels of socio-economic development to enhance the external validity of the findings, and the implementation of subgroup analyses based on tumor type (oesophageal versus gastric cancer) as well as tumor stage and treatment modality (surgical versus non-surgical) to identify epidemiologically related healthcare service disruptions that may have a differential impact. In addition, more detailed co-morbidity analyses, including severity grading and disease-specific classifications, were conducted to gain insight into how co-morbidities may have affected patterns of use of inpatient oncology services in the UGI. Such studies would help to determine whether the trends we observed, including shorter lengths of stay, changes in cost structure, and differences in occupation, social security, and co-morbidities among hospitalized populations, are consistent across population characteristics, geographic distribution, and healthcare resource conditions.

## Conclusion

Our study shows that the outbreak led to a reduction in the use of UGI tumor inpatient services in Yangzhou City due to policy, hospital operations, and individual patient choice. The COVID-19 pandemic exacerbated the disease burden among patients with UGI tumors, with significant increases in self-payment expenses. During the outbreak, hospital days and total hospitalization costs decreased instantaneously, yet in the post-pandemic period, both hospital days and expenses surged, surpassing pre-pandemic levels by late 2021. The structure of medical costs also changed, with notable increases in the cost of blood and consumables. Additionally, there were significant changes in patients’ occupational structure, health insurance utilization, and complication status.

To address these issues, we must strengthen emergency management and implement precise prevention and control measures based on epidemic dynamics. Ensuring the safe supply of blood products and emergency medical supplies is crucial. We need to enhance the primary healthcare institution system to guarantee patients’ access to medical services. Given that the COVID-19 pandemic may exacerbate health inequalities, optimizing the medical insurance system, providing targeted subsidies to medical institutions, curbing the growth of unreasonable medical costs, and offering special protection for vulnerable groups are essential steps. This research on the impact of public health emergencies on the utilization of inpatient cancer services provides valuable insights for policymakers, highlighting the need to continuously work on reducing inequities in the use of inpatient services during emergencies.

## Data Availability

The data analyzed in this study is subject to the following licenses/restrictions: the dataset presented in this article is not readily available because it involves the patient’s personal privacy. We signed an agreement to ensure that the data will not be leaked and transmitted. Requests to access these datasets should be directed to luhui@njmu.edu.cn.
